# The Impact of Microelectrode Pattern on the Sensitivity of Tracing Environmental CO_2_ Deficiency in Cellular Metabolism by a New Design of Electrochemical Biosensor

**DOI:** 10.3390/bios13080762

**Published:** 2023-07-27

**Authors:** Faegheh Bourbour, Hamed Abadijoo, Fatemeh Nazari, Hamideh Ehtesabi, Mohammad Abdolahad

**Affiliations:** 1 Nano Electronic Center of Excellence, Nano Bio Electronic Devices Laboratory, School of Electrical and Computer Engineering, University of Tehran, Tehran 1439957131, Iran; 2 Nano Electronic Center of Excellence, Thin Film and Nano Electronics Laboratory, School of Electrical and Computer Engineering, University of Tehran, Tehran 1439957131, Iran; 3 Institute of Cancer, Imam Khomeini Hospital, Tehran University of Medical Sciences, Tehran 1416634793, Iran; 4 UT and TUMS Cancer Electronics Research Center, Tehran University of Medical Sciences, Tehran 1416634793, Iran; 5 Faculty of Life Sciences and Biotechnology, Shahid Beheshti University, Tehran 1983969411, Iran

**Keywords:** cell secretion, chip pattern, cyclic voltammogram, cell culture solution pH, hypo-CO_2_

## Abstract

Here, two different electrode patterns are described as cyclic voltammetry (CV) biosensors to detect the effect of a hypo CO_2_ condition (for 6 h) in ambient on cellular secretion. The cells were selected from breast cancer and endothelial standard lines. Changes in CV peaks of the secretions were recorded by the modified pattern whereby increasing the interactive surface with homogenous electric paths was considered by simulation before fabrication. The results of the simulation and experimental procedures showed a meaningful correlation between hypo CO_2_ samples and the occurrence of CV oxidative peaks at about 0.07 V and reductive peaks at approximately −0.22 V in the modified biosensor in all cell lines, while no apoptosis was found in any of the control and hypo CO_2_ samples. This observation could not be related to the lack of H^+^ (alkaline pH induction) in the media solution as such peaks were not observed in the pure cell culture medium but had been maintained in the hypo CO_2_ ambient. This approach could be used as a cell-free sensor to monitor ambient shocks. This may not induce apoptosis but may be vital in the proliferation and protein expression of the cells, such as the hypo CO_2_ ambient. The sensor is not disposable in use and showed repeatable responses after rinsing.

## 1. Introduction

Electrochemical analysis of cellular secretions has become more attractive due to its valuable extractable data, non-invasive sample preparation, and simple operation. These tests can be carried out without inducing any perturbation in standard assays. Cellular metabolism can be traced by monitoring the microenvironment secretions by constantly sensing chemical cues such as H_2_O_2_ and converting them into biochemical signals using electrochemical biosensing methods like cyclic voltammetry and electrochemical impedance spectroscopy [1,2,3,4,5]. In this regard, much research has been performed to obtain substantial amounts of information and trace the condition of cells. These processes allow us to check cellular responses and adaptations due to changes in their vital states or their micro-environment parameters [1,6,7].

This may be helpful in acquiring an early warning about crucial cellular functions such as hypermetabolism, mitosis, or apoptosis.

Understanding the mechanisms underlying each of these pathways is complex and requires a plethora of molecular and proteomics analyses. Nevertheless, the combination of electrochemical science, surface chemistry, and biological evidence may be beneficial in tracking biological evidence by a uniquely recorded electrochemical signal from the cellular environment.

One of the conditions that can affect cell secretion is depriving the cells of CO_2_ during cell proliferation [8,9].

One of the methods to obtain cell differentiation in various conditions is to change their normal culturing situation and then examine the differences in some characteristics. In some cases, to shock the cell and observe the ensuing effect on behavior and possibly cellular responses, the amount of serum (Fetal Bovine Serum), glucose [10,11,12,13], temperature, or CO_2_ [14] of the standard cell-culture solution has been changed, and then some differences or some symptoms as the result of these changes have been compared. Here we discuss the design of a microchip with an engineered electrode pattern to find any specific electrochemical spikes in correlation with cellular secretion in a reduced CO_2_ environment. The influence of reduced CO_2_ environment parameters and the effect of the electrode pattern in sensing these shreds of evidence are discussed in this paper. Various types of normal and cancer cell lines were tested by this system, and the trace of their CO2 and the reduction of related derivatives on electrochemical sensing of their secretion was evaluated.

This research may shed new light on cell-free monitoring of cellular metabolism and provide early warning about any problems that may happen in cellular incubation, possibly causing changes in cell secretion without direct intervention on the cells or using flow cytometry.

## 2. Materials and Methods

### 2.1. Cell Culture

Human umbilical-vein endothelial cells (HUVEC), MDA-MB-231, and MCF-7 as human metastatic breast cancer cell lines were all purchased from the National Cell Bank of Pasteur Institute of Iran (NCBI, Tehran, Iran). The considered cell lines (MDA-MB-231, MCF-7, HUVEC) were thawed in a cell culture flask. After a week, when the confluency of the cells in the flask had become about 80%, every cell line was shared in 6-well petri dishes (about 20,000 cells in each well). Corresponding cells were cultured in 4 milliliters of cell-culture solution containing 94% Dulbecco’s Modified Eagle Medium (DMEM, Gibco) complemented with 5% fetal bovine serum (FBS, Bioceramed, Tehran, Iran) and 1% penicillin/streptomycin (Bioceramed, Tehran, Iran). A manual cell-counting method using a hemocytometer Neubauer was used to measure the quantity of the cells in the flask and wells of the petri dish. The petri dish was kept in the normal situation in a CO_2_ incubator at 37 °C with 97% humidity and 5% CO_2_ for five days.

**Figure 1:**
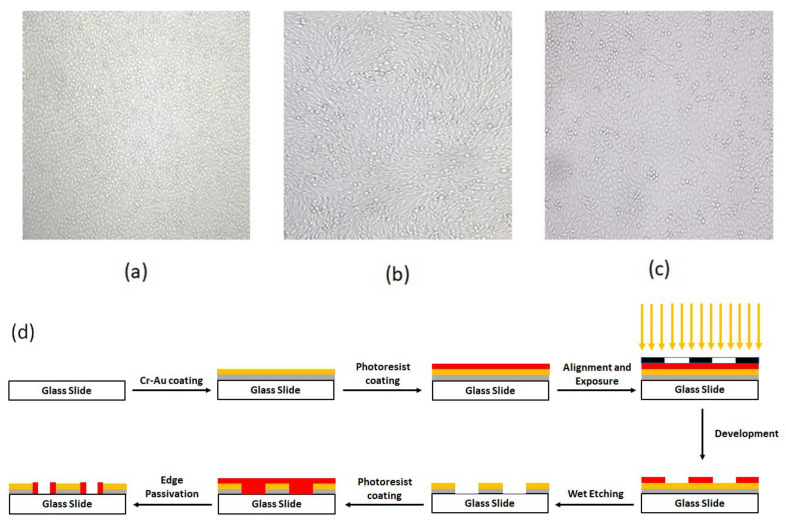
The confluency of (**a**) HUVEC, (**b**) MDA-MB231, and (**c**) MCF-7 cells (**d**) schematic of detail of chip fabrication.

After five days and at the time that the confluency of the cells reached near 90%, for the normal proliferation condition, 50% of the cell culture solution was collected for electrochemical measurements. The confluency of the cells at the time of the test is shown in Figure 1a–c. For our CO_2_ reduction condition, the petri dish was put outside the incubator for 6 h, and then the rest of the cell-culture solution of all wells of the petri dish was tested in a separate process. Finally, the cyclic voltammograms of these two conditions (proliferation and apoptosis) were compared, using both chip patterns. In addition, the pH value of both solutions (normal cell culturing and CO_2_ reduced culturing solution) was measured with an electrical pH meter (Metrohm, Herisau, Switzerland).

### 2.2. Device Fabrication

#### 2.2.1. Fabrication of the Chip

The fabrication process started by coating the glass with 60 nm Cr and then 100 nm Au by RF sputtering. The base pressure for the sputter deposition was adjusted to 5 × 10^−6^ mBar. After the base pressure was obtained, argon plasma was utilized to commence the deposition. The deposition pressure and power were adjusted to 2.5 × 10^−2^ mBar–100 W, and 1.8 × 10^−2^ mBar–250 W for Cr and Au deposition, respectively. The thickness of the deposited layers was monitored during the deposition process using piezoelectric crystal oscillators inside the chamber. After the completion of the deposition process, a positive photoresist (Shipley s1813) was spin-coated on the slide’s surface for 30 s at 3000 RPM. Then, the slides were prebaked for 8 min at 110 °C before exposure. The alignment and exposure processes were performed using a mask aligner (Karl Suss MA6 mask aligner, Garching, Germany). After exposure, the slides were developed and then post-baked for 18 min at 90 °C. Finally, the patterning process was completed via performin wet etching of Au and Cr layers. To electrically passivate the Au/Cr edges, the steps of photolithography were repeated by the reverse pattern. Figure 1d depicts these lithography process steps.

#### 2.2.2. Precleaning of the Device before the Test

Before every test, the device was rinsed with DI water, and the container was filled up to 70% volume with DI water. Then, the chip surface was cleaned with a mild ultrasonic wave of 5-W power with its tip 5 mm above the chip surface for 1 min to remove every residue that may have remained from the previous test.

### 2.3. Viability Test

To test the viability of the cells, Annexin V/PI flow cytometry and AO/PI (Acridine Orange/Propidium Iodide) staining assays were performed. As we know, the extent of AnnexinV and PI binding in the cells is related to the stage of their viability [15,16,17,18]. The test was done for three cell lines (MDA-MB-231, MCF-7, and HUVEC) in the normal culturing situation and for the cells that had been kept out of the incubator for 6 h. The standard AO/PI staining protocol was used [19]. Acridine Orange (AO) is a dye with fluorescent characteristics that all cell nuclei can absorb. On the other hand, propidium iodide (PI) is also another dye with fluorescent properties, and its expression shows membrane rupture [20]. The test was performed for the cell lines mentioned above, both in the normal culturing condition (5% CO_2_, 37 °C) and after 6 h outside the incubator.

### 2.4. Cyclic Voltammetry

Today, electrochemical methods have the advantages of simplicity and low cost; as well, they provide information about the presence of some compounds in the medium. Thus, this area has attracted much attention in many fields of research, such as biology. Cyclic voltammetry is extensively used for electrochemical sensing in biological media [21,22,23,24,25].

To obtain the electrochemical characteristics of cellular secretion, after the cell culturing procedure had been accomplished as explained in Section 2.1, 2 milliliters of the cell culture medium of each cell line (neither diluted nor pre-processed) was poured on the sensor, and the voltammogram was measured using both sensors. The response was measured using a portable electrochemical analyzer (IVIUM, Compact stat.h, Ivium Technologies, Eindhoven, The Netherlands) in three-electrode cyclic voltammetry (CV) mode. The measurement was performed with DC sweeping voltage from −800 mV to 800 mV, and the scan rate was set to 50 mV/s.

## 3. Results and Discussion

Figure 1a–c shows the confluence of the cultured cell lines and their vital appearance as an indicator for the desired parameters of culturing media, such as supplemented materials and ambient humidity, as well as CO_2_ and related derivatives.

To test the effect of hypo CO_2_ on cellular secretion, we designed a cyclic voltammetric electrochemical sensor with two different electrode patterns (commercial CV electrodes with circular-work electrodes and spiral-interdigital electrodes) as described in the methods section. The schematic of these steps is shown in Figure 1d.

In order to achieve better results and also to obtain the benefits of both interdigital and commercial patterns of electrochemical probes, we used the combination of two common patterns named spiral-interdigital electrode. The advantage of the interdigital pattern is that the area of the uniform electric field is more than in other patterns; it is also adjustable and designable, as with different numbers and different lengths of interdigits to obtain different properties. However, this pattern also has some unfavorable properties such as sharp corners and an edge effect that causes some non-uniform electric field pattern, which can affect the measurement outcome and produce some undesirable results. Due to the circular shape of the commercial pattern, this design has the advantage of a uniform electric field without any corner and edge effects. Nevertheless, this pattern also has some disadvantages, such as constant design and a small area of the uniform electric field, compared to interdigital design. To reduce these problems and produce a design with better properties, we propose a spiral interdigital pattern with the advantages of these two abovementioned patterns but with reduced problems. From the Cottrell Equation (Equation (1)), it is known that the measured oxidation/reduction current is proportional to the electrodes’ effective surface area and the concentration of the relevant reactants in the vicinity of the corresponding electrodes [26]. This phenomenon can be harnessed to increase the sensitivity of the electrochemical sensors operating on electric current measurement. Spiral–interdigital electrodes not only maintain a uniform electric field in the measurement region but also enhance the sensitivity of the measurement by increasing the effective surface area.

(1) $$i\left(t\right)=\frac{\mathrm{nFA}c_{j}^{0}\sqrt{D_{j}}}{\sqrt{\pi t}}$$

In the Cottrell Equation (above), *i*(*t*) stands for the current in amperes, *n* is the number of electrons, *F* is the Faraday constant (96,485 columb/mol), *A* is the area of the (planar) electrode in cm^2^, $c_{j}^{0}$ is the initial concentration of the reducible analyte *j* in mol/cm^3^, $D_{j}$ is the diffusion coefficient for species *j* in cm^2^/s, and *t* is the time in seconds.

**Figure 2:**
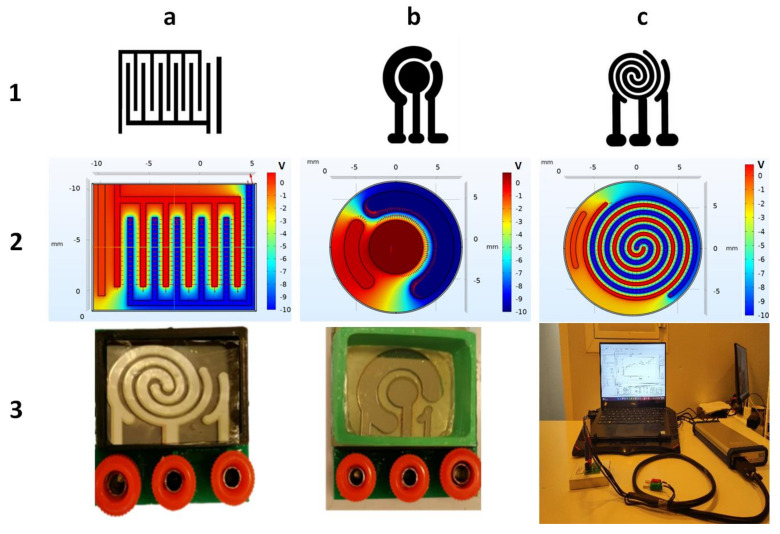
(**a-1**) Interdigital, (**b-1**) commercial, and (**c-1**) spiral-interdigital patterns and simulation of electrical fields and potentials of (**a-2**) inter-digital, (**b-2**) commercial, and (**c-2**) spiral-interdigital design by COMSOL Multiphysics software and last set-up that has been fabricated. (**a-3**) Spiral-interdigital chip, (**b-3**) commercial chip. (**c-3**) Test set-up, respectively.

The apparent differences between these three patterns are shown in Figure 2.

The spiral-interdigital pattern shows promising results in some cases, such as in the procedure that will be explained in this paper. The distribution of the electric field and its potential in these three patterns (commercial, interdigital, and spiral-interdigital) were simulated by COMSOL Multiphysics software and are shown in Figure 2.

As we have shown in these simulations, in the spiral-interdigital pattern, the area where a uniform electric field can be achieved is significantly more than others, and it can be designable with more circulations. With this pattern, we have also reduced the edge effect to the minimum amount and near zero.

To reduce the edge effect of the Au-Cr layer on the glass slide, the Au-Cr edge was passivated by a photoresist, and to make the photoresist biocompatible, it was baked at 180 °C for 1 h without direct air contact. This step is shown in Figure 1d schematically.

**Figure 3:**
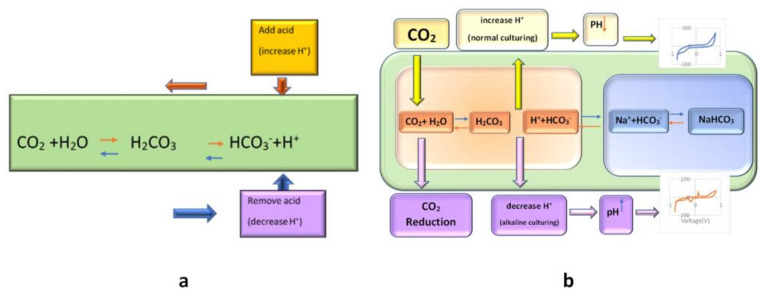
(**a**): The principle of bicarbonate buffering (according to Le Chatelier’s principle, pH will be stabilized by an appropriate amount of CO_2_% and in accordance with the buffering property of carbonic acid and its conjugate base); (**b**): schematic of the effect of CO_2_ reduction on the cell medium and its result on the CV of cell culture solution.

The utilized setups that were fabricated and used in this procedure are shown in Figure 3. Both electrochemical biosensing platforms consist of 3 electrodes (working, counter, and reference electrodes). All three electrodes were fabricated using Cr-Au sputtering deposition and a standard photolithography process. In these setups, the right port is connected to the reference electrode, the middle port is connected to the working electrode, and the left port is connected to the counter electrode.

From the evidence, it seems that cells will secrete different materials in different situations. Thus, we can apply different conditions to the cells and test their responses to these conditions.

In most cell-culture incubators, the CO_2_ level is set between 5–10%. CO_2_ has no metabolic role in cell culture. Its purpose is to dissolve into the cell culture medium and react with its water to form carbonic acid. As it has the buffering role, with its conjugate base (the dissolved bicarbonate ions in the medium, HCO_3_^−^), it stabilizes the pH of the medium [27,28,29,30].

Bicarbonate buffering works through Le Chatelier’s principle. Increased acidity in the medium is manifested by an increase in hydrogen (H^+^) ions; free bicarbonate ions then react with the extra H^+^ ions to form carbonic acid, “shifting the reaction to the left”, stabilizing pH (see Figure 3). Similarly, a decrease in H^+^ ions will result in a “shift to the right”. If we remove the CO_2_ from the ambient, the CO_2_ would be reduced, and subsequently, Le Chatelier’s balance reaction would shift to the left, and further reaction of H^+^ with HCO_3_^−^ results in reduced H^+^ and the increased pH of the ambient (shift of reaction to left); hence the pH of the media would not be neutralized by bicarbonate buffering. Thus, an increased pH related to the electrochemical peak would appear (Figure 3). This peak appearance could be traceable after 6 h of CO_2_ inhibition by our proposed biosensor. Other data about the increase in the CV peak of the active basic solution by increasing the pH are in complete correlation with our result. This peak may be related to the cell secretion that the cells may secrete in the media in this new condition (alkaline situation with reduced CO_2_).

In this paper, we examine the effect of CO_2_ reduction and, as a result, pH elevation on the cyclic voltammogram of the cell secretion recorded separately by two different sensor patterns. The schematic protocol of this research is shown in Figure 3.

**Figure 4:**
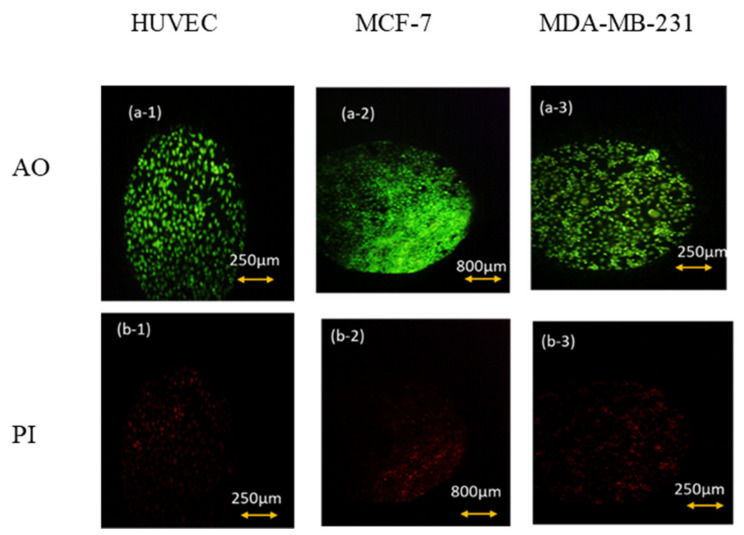
AO/PI viability test for (1) HUVEC, (2) MCF-7. (3) MDA-MB-231 cell lines after 6 h of keeping cells out of the incubator. (**a**) AO fluorescent images that show live cells’ density and (**b**) their corresponding PI fluorescent images that represent the density of dead ones.

**Figure 5:**
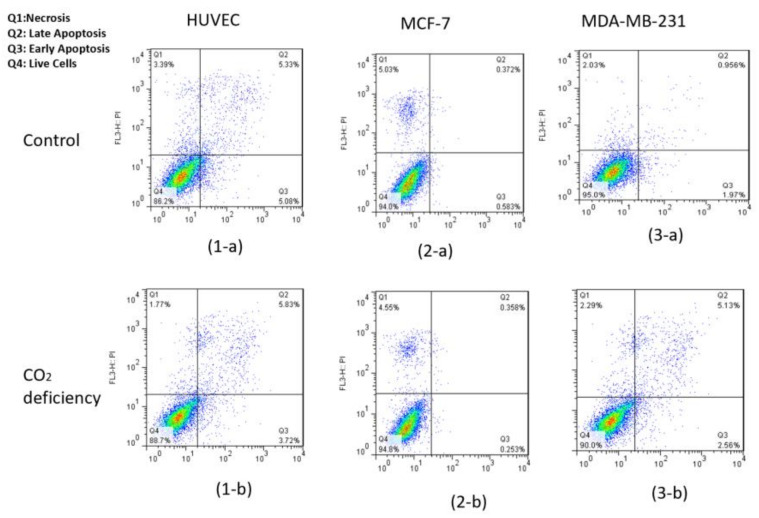
Results of flow cytometry of Annexin-V & PI test for (1) HUVEC, (2) MCF-7, and (3) MDA-MB-231 cell lines (**a**) before and (**b**) after 6-h CO_2_ deprivation, respectively.

As can be seen in Figure 4, after 6 h of keeping the cells out of the incubator, the amount of AO absorbed in the cell nucleus is much higher than PI. Thus, we can conclude that keeping cells out of the incubator for 6 h does not significantly affect the amount of necrotic and CO_2_-reduced cells. This conclusion is also confirmed by the flow cytometry test and by the results shown below in Figure 5 and Table 1.

Also, flow cytometry results indicated that the maintenance of the cells had been incubated in the hypo CO_2_ ambient in a viable state.

**Figure 6:**
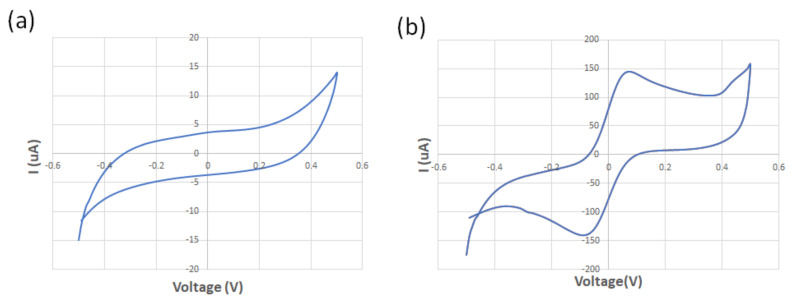
The obtained voltammogram of the (**a**) DI water; (**b**) 125 μM of standard redox probe (K3[Fe(CN)6]).

The spiral-interdigital sensor was characterized using cyclic voltammetry at a scan rate of 100 mV/s and using 125 µM of standard redox probe (K3[Fe(CN)_6_]) and DI water at room temperature. While no oxidation/reduction peaks were observed in the voltammogram of the DI water, the obtained voltammogram confirmed the existence of the oxidation/reduction peak regarding the redox probe solution. These results show the promising operation of the spiral-interdigital electrode (Figure 6). Perhaps adding EIS analysis near CV could provide more reliable data with better analysis, which may be a limitation of this research and must be covered in the future.

**Figure 7:**
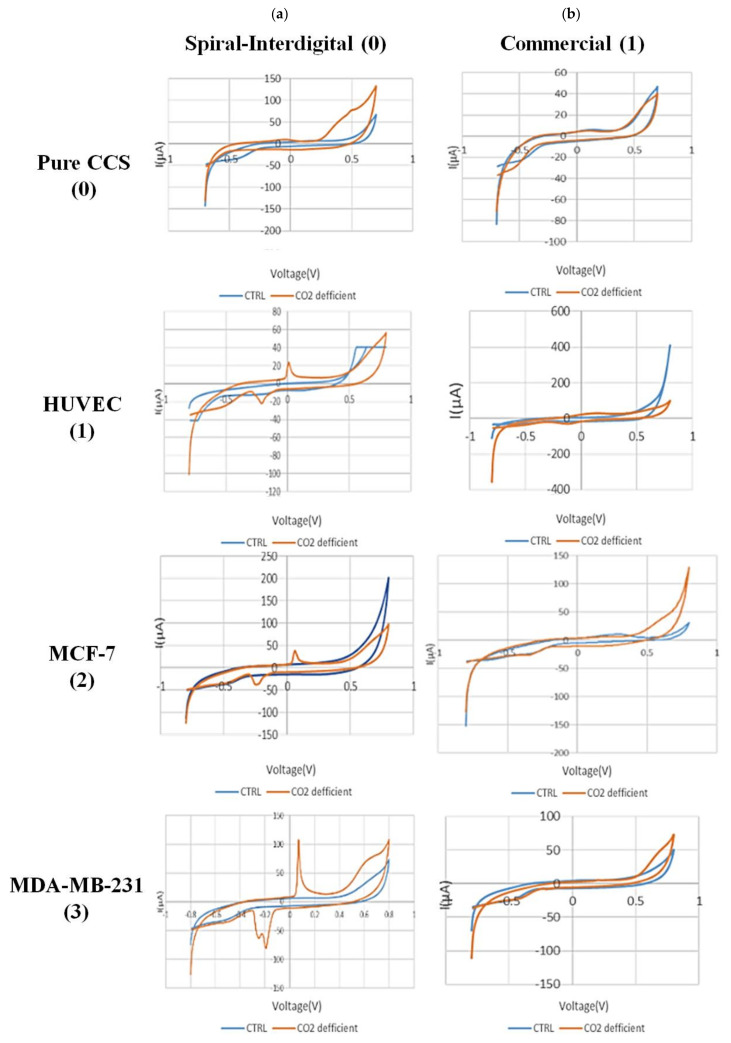
Comparison of cyclic voltammograms (CV) of cell-free cell-culture solution of (0) pure cell-culture solution, (1) HUVEC, (2) MCF-7, and (3) MDA-MB-231 cell lines in normal (blue line) and hypo CO_2_ (orange line) situation by (**a**) spiral interdigital and (**b**) commercial pattern chips, respectively.

Figure 7 shows the CV results of secretion media from all types of experimented cells in normal and hypo CO_2_ ambient with both conventional and spiral-interdigital electrodes.

The CV scans were swept from −0.8 V to 0.8 V in the supernatant of the cells at a rate of 50 mV/s. As shown in Figure 7(a-1), a pair of well-defined peaks for some redox agents were just observed at the spiral-interdigital-shaped micro-electrode, with an oxidative peak potential around 0.07 V and following reductive peak potential between −0.2 V and −0.24 V for MDA-MB-231 cell line. As well, in Figure 7(a-2), these peaks were seen near 0.06 and about 0.25, respectively, for MCF-7 cell line; in Figure 7(a-3), the peaks were located near 0 and near 0.23, respectively, for the HUVEC cell line. These peaks also have no significant differences for different cells, and we can put these results in a nutshell and conclude that for all of these three cell types, the oxidative peak is around 0 V and its related reductive one is around 0.22 V. In contrast, these peaks of apoptosis-relation events were not detected on the CV curves of conventional circular micro-electrodes. Both decorations of the microelectrodes detected no peaks for the media solution of cancer cells that had been kept in an incubator (Figure 7((a-2),(b-2))). This means that the appearance of peaks in just spiral-interdigital-shaped microelectrodes after interaction with media of extra incubator cells should be just related to CO_2_ reduction shock. Also, an increasing trend was observed in the measured oxidative and reductive currents for HUVEC, MCF-7, and MDA-MB-231, respectively. It may be concluded that more malignant cell lines were observed to result in greater oxidative and reductive currents at corresponding peaks.

Figure 2 shows the interactive surface and field distribution of conventional circular and spiral-interdigital-shaped microelectrodes obtained by software simulation (COMSOL Multiphysics 5.4). The simulation result depicts that the spiral-interdigital-shaped electrodes applied much better electric field uniformity over the entire surface and formed a well-organized interactive surface distribution and site bonding by the solution under the biased voltages.

As mentioned before, in order to monitor the pH of the solution before and after the CO_2_ reduction culturing situation, the value of this parameter was measured in both cases, and as expected, the pH value in the condition of 6 h after the reduction of CO_2_ was increased approximately 1.5 to 2 units.

With these cues, we can also conclude that because of CO_2_ deficiency in the cell medium, some signaling pathways would be triggered in the cells. These pathways may be the result of pH changes in the medium. As a result of these processes, some chemical substances will be secreted by the cells in the medium. These substances, which are still unknown to us, play a key role in the recorded peaks (Figure 7a). It is worth noting that the peak formation is not directly due to CO_2_ deficiency and pH increment in the media itself. As seen in Figure 7(a-0), such peaks were not found in the same situation in pure DMEM + FBS + PenStrep media that had been maintained out of the incubator for 6 h.

In brief, achieving shock-related (such as hypo CO_2_ situation) responses of the cells from the environmental bio-interfaces requires a well-established recording of ambient mechanical, biochemical, and bioelectrical signals in real time. Among these approaches, electrochemical sensors showed powerfully advanced and promising responses to enable a better understanding of the biological evidence because chemical agent releasement is the main indicator for cellular functions. On the other hand, electrical compiling is the fastest procedure with the smallest number of mistaken analyses [29]. We provide insight into tracing the secretion by electrochemical approaches by distinctive sensor patterns to increase the interactive bio-surface to find meaningful data in correlation with CO_2_ reduction. Electrochemical data recording from two different patterns of sensing electrodes and the effect of the pattern on better sensation elaborated the impact of sensor mechanism and pattern in this field of research.

## 4. Conclusions

In summary, we showed the effect of electrode patterns in cyclic voltammetry-based detection of hypo CO_2_ perturbation on cellular metabolism by recording data from the media secretion. The electric field and current distribution in correlation with the interactive surface between electrode and electrolyte seem to play roles in this observation. The peak is related to the cells’ metabolism changes, which may secrete unknown agents. No peaks were observed in the DMEM media solution that had been kept in hypo CO_2_ ambient. Thus, the reason for peak appearance may not be directly due to the pH increment of the media in CO_2_ deficiency. Hence, this situation may either affect the ionic secretion of the cells or induce perturbation in the balance of Le Chatelier’s principle. This may be a fast cell-free assay to observe the hypo CO_2_ trace in cell culture. Also, such sensors may be helpful in the study of similar pieces of evidence in diverse 3D culture formats, including spheroids. As electrochemistry could provide the requirements to accomplish this goal, parameters other than electrode pattern, such as electrode materials (e.g., conducting polymers) or functionalizing agents (e.g., increasing hydrophobic properties), are interesting building blocks for the creation of electrically responsive real-time biomonitoring.

## Figures and Tables

**Table 1:** Quantitative analysis of the Annexin V and PI test for HUVEC, MCF-7, and MDA-MB-231 cell lines for control and hypo-CO_2_ conditions.

Cell Type & Condition/Vitality	Live%	Early Apoptosis%	Late Apoptosis%	Necrosis%
HUVEC (Ctrl)	86.20	5.08	5.33	3.39
HUVEC (CO_2_ deficiency)	88.70	3.72	5.83	5.83
MCF-7 (Ctrl)	94.00	0.58	0.37	5.03
MCF-7 (CO_2_ deficiency)	94.80	0.25	0.36	4.55
MDA-MB-231 (Ctrl)	95.00	1.97	0.96	2.03
MDA-MB-231 (CO_2_ deficiency)	90.00	2.56	5.13	2.29

## Data Availability

Datasets are available upon request.
